# Mucormycosis in COVID-19 Patients: A Case-Control Study

**DOI:** 10.3390/microorganisms10061209

**Published:** 2022-06-13

**Authors:** Awadh Kishor Pandit, Poorvi Tangri, Shubham Misra, Madakasira Vasantha Padma Srivastava, Sushma Bhatnagar, Alok Thakar, Kapil Sikka, Smriti Panda, Venugopalan Y. Vishnu, Rajesh Kumar Singh, Animesh Das, Divya M. Radhakrishnan, Achal Kumar Srivastava, Rajeshwari Subramaniam, Anjan Trikha, Ayush Agarwal, Roopa Rajan, Vibhor Upadhyay, Sathish Parikipandla, Anup Singh, Arvind Kairo

**Affiliations:** 1 Department of Neurology, All India Institute of Medical Sciences, New Delhi 110029, India; poorvi.tangri2@gmail.com (P.T.); shubham.misra30@gmail.com (S.M.); vasanthapadma123@gmail.com (M.V.P.S.); vishnuvy16@yahoo.com (V.Y.V.); drrajeshrims679@gmail.com (R.K.S.); animeshdas05@gmail.com (A.D.); dr.divyamr@gmail.com (D.M.R.); achalsrivastava@hotmail.com (A.K.S.); ayushthetaurian@gmail.com (A.A.); roops84@gmail.com (R.R.); docvibhor@gmail.com (V.U.); satish.svmc2k86@gmail.com (S.P.); 2 Department of Onco-Anesthesia, Pain and Palliative Care, All India Institute of Medical Sciences, New Delhi 110029, India; shumob@yahoo.com; 3 Department of Otolaryngology and Head-Neck Surgery, All India Institute of Medical Sciences, New Delhi 110029, India; drathakar@gmail.com (A.T.); kapil_sikka@yahoo.com (K.S.); smriti.panda.87@gmail.com (S.P.); anoop.aiims1@gmail.com (A.S.); orlhns@aiims.edu (A.K.); 4 Department of Anesthesiology, Critical Care and Pain Medicine, All India Institute of Medical Sciences, New Delhi 110029, India; drsrajeshwari@gmail.com (R.S.); anjantrikha@gmail.com (A.T.)

**Keywords:** mucormycosis COVID-19, intracranial fungal infection, COVID-19

## Abstract

(1) Background: During the second wave of COVID-19, India faced a rapid and sudden surge of not only COVID19-delta variant cases but also mucormycosis, making the infection even more fatal. We conducted a study to determine factors associated with the occurrence of mucormycosis in patients with COVID-19. (2) Methods: This case–control study comprised 121 patients; 61 cases (mucormycosis with COVID-19) and 60 controls. Patients were included from April 10, 2021 onwards. Follow-up was conducted after about 90 days and health status was recorded based on the modified Rankin Scale (mRS). (3) Results: Mucormycosis with COVID-19 cases had a median (IQR) age of 49 (43–59) years with 65.6% males and were older (95% CI 1.015–1.075; *p* = 0.002) than in the control group with median (IQR) 38 (29–55.5) years and 66.6% males. Baseline raised serum creatinine (OR = 4.963; 95% CI 1.456–16.911; *p* = 0.010) and D-dimer (OR = 1.000; 95% CI 1.000–1.001; *p* = 0.028) were independently associated with the occurrence of mucormycosis in COVID-19 patients. Additionally, diabetes mellitus (OR = 26.919; 95% CI 1.666–434.892; *p* = 0.020) was associated with poor outcomes and increased mortality in patients with mucormycosis with COVID-19 as per the multivariable analysis. A total of 30/61 mucormycosis patients had intracranial involvement. (4) Conclusions: The study observed elevated levels of baseline raised creatinine and D-dimer in mucormycosis pa-tients with COVID-19 as compared to the control group. However, future studies may be conducted to establish this cause–effect relationship.

## 1. Introduction

Fungi belonging to the order Mucorales cause mucormycosis. Although *Rhizopus arrhizus* is the most common etiological agent of mucormycosis in India, infections due to *Rhizopus microsporus*, *Apophysomyces variabilis, Rhizopus homothallicus, Mucor* spp., etc., are increasingly reported [1,2]. The prevalence rate of mucormycosis in India is about 80 times higher than in developed countries during the pre-COVID time. Reports state that on average, per every 1000 people, 0.14 cases are found [3]. During the second wave of COVID-19, India suffered from a catastrophic outburst of cases and a rapid transmission of the disease due to the highly infectious delta strain (B.1.617.2). A research letter stated that India reported 28,000 cases of mucormycosis by 7 June 2021 and observed the occurrence of stroke in patients with COVID-19-associated mucormycosis infection. India is one of the leading nations in having a higher proportion of the population inflicted with diabetes mellitus. Well-established parameters that may lead to the occurrence of mucormycosis are the availability of free iron, high incidence of diabetes mellitus, ketoacidosis, and immunosuppression [4]. Gujarat is the state with the highest number of mucormycosis cases (3726) in recovered and COVID-19 infected people. An increased number of cases were observed in states such as Rajasthan, Haryana, Uttarakhand, Karnataka, Andhra Pradesh, Madhya Pradesh, and New Delhi [5]. As a consequence of the outburst of mucormycosis throughout the nation, it was declared an epidemic in several states and was declared a notifiable disease by health authorities [6].

Patients infected with the COVID-19 delta strain underwent prolonged hospitalizations, suffered from severe symptoms, and as a result, had a weakened immune system. The predisposing factors that contributed to this rapid surge of mucormycosis cases during the COVID-19 pandemic are thought to be co-morbidities such as hyperglycemia, hypertension, administration of steroids, and immunocompromised health of patients due to COVID-19 or other underlying health conditions [5,7]. A study reported that high levels of ferritin in patients also act as a contributing factor toward the manifestation of mucormycosis in immunocompromised patients [8]. High levels of iron, an essential element for fungal growth, act as a source for the development and cellular growth of causative agents [9].

Mucormycosis causing fungi are most commonly found in decaying organic matter and infect humans through nasal inhalation and subsequently involve paranasal sinuses. Uncommon manifestations of cranial invasion include sagittal sinus thrombosis and epidural and subdural abscess [10]. Meningitis is rare, but when present, it may manifest as obstructive hydrocephalus due to infiltration of the ventricular lining [11]. Mucorales fungal infection due to duration of infection, the severity of disease, and host immunity invade host system. PNS, orbital apex, bone erosion, angioinvasion, and the systemic involvement of fungal hyphae disrupts the endothelium [12]. Various forms of clinical manifestations in patients with mucormycosis include: pulmonary, gastrointestinal, cutaneous, and rhino-orbital-cerebral [13].

We have conducted this case–control study which aims at understanding the factors that may have led to the occurrence of mucormycosis in COVID-19 patients in the Indian population. It further highlights the factors that may have led to intracranial involvement of mucormycosis in the COVID-19 patients.

## 2. Materials and Methods

This case–control study was conducted following the approval from the Institute Ethics Committee (IEC-426/02.07.2021, RP-44/2021). The study was conducted at COVID-19 converted center of All India Institute of Medical Sciences (New Delhi) named National Cancer Institute (NCI), AIIMS, Jhajjhar a tertiary care referral hospital with a separate COVID-19 ward and about 25 ICU beds [14]. Patients included in the study were mostly from various parts of Haryana, New Delhi, and surrounding areas. Following valid consent, all consecutive cases admitted with mucormycosis (laboratory-confirmed) were included in an ambispective (prospective and retrospective) manner. Cases and controls included in the study were from 10 April 2021 onwards during the second wave of COVID-19. Date of enrolment of the last patient in the study was 31 May 2021. Patients were included in the control group if they were asymptomatic for mucormycosis and normal radiology [normal Computed Tomography (CT) of PNS and head]. The demographic, medical history, comorbidities, treatment received during and before hospitalization, and long-term clinical outcomes (at follow-up: treatment, clinical outcome, modified Rankin Scale (mRS) [15], and status of co-morbidities) were recorded. Follow-up was performed telephonically after a time period of about 90 days. In case of unavailability or inability of the patients, their next of kin were approached for follow-up status. Poor outcome was defined as mRS score of 3 to 6. All the patients received injection of amphotericin B (1 mg of amphotericin B IP/USP in liposomes, suspended in normal saline) at a dose of 5–10 mg/Kg body weight daily up to a duration of four to six weeks along with a tablet Posaconazole at a dose of 300 mg twice a day for two days followed by once daily until about three to six months and adjunct surgery (where indicated). Additionally, radiological, blood investigations, microbiological and histopathological data were recorded for both patients and controls. The standard care to the patients did not interfere. The non-COVID-19 patients or controls not consenting were excluded from the study.

The diagnosis of COVID-19 was based on positive RT-PCR (reverse transcriptase PCR) for SARS-CoV-2 or rapid antigen test [16] on nasopharyngeal or oropharyngeal swabs. Diagnosis of mucormycosis was confirmed by potassium hydroxide (KOH) staining of tissue obtained by nasal, paranasal, paranasal sinuses or palate and histopathology of the tissue or culture. However, dissemination of mucormycosis infection was not considered unless patients had related symptoms. Computed tomography (CT) scans of lungs and abdomen were performed in limited cases if patients had related pulmonary or gastrointestinal symptoms. Intracranial involvement was based on involvement of dura mater and beyond on the basis of neuroimaging (CT/MRI). Patients infected consecutively with mucormycosis within a time period of 60 days after testing positive for COVID-19 were included in the study. The following parameters were considered: C-reactive-protein (CRP) was considered to be elevated if it was >6 mg/L; procalcitonin was considered elevated if it was >0.07 ng/mL; D-dimer was considered elevated if it was >232 ng/L; absolute lymphocyte count was considered low if it was <1500 per mm^3^; ferritin was considered elevated if it was >150 ng/mL and hemoglobin A1C (HbA1C) was considered elevated if it was >6.4 % (as diabetes mellitus).

### Statistical Analysis

After the collection and compilation of data, the baseline characteristics of all the patients were tabulated. To determine the spread of mucormycosis infection amongst the COVID-19 patients, analysis of the collected data was performed followed by determining the factors associated with intracranial extension. The normal distribution was checked using the Shapiro–Wilk test. If normally distributed, the continuous variables were represented by mean with standard deviation (S.D.), and the difference between the two groups was assessed using the student *t*-test; whereas, if non-normally distributed, the continuous variables were represented by median (interquartile range, IQR) and the difference was assessed using Mann–Whitney U test. The dichotomous variables were represented by number (percentage) and the difference between the two groups was calculated using Chi-square test or Fisher exact test (if values <5). Variables with *p*-value < 0.1 in the univariable analysis were subjected to forward stepwise multivariable logistic regression analysis to frame the prediction models for assessing the independent association of mucormycosis with 90-day poor outcome and 90-day mortality compared to control subjects. A subgroup analysis was conducted between mucormycosis patients showing intracranial involvement and non-intracranial mucormycosis cases. The area under the receiver operating characteristic (ROC) curve (AUC) along with sensitivity, specificity, positive predictive value (PPV), and negative predictive value (NPV) was determined for each prediction model. The multicollinearity between the predictor variables was tested using the variance inflation index (VIF) and variables with a VIF value >2.5 were considered highly correlated and were subsequently removed from the model. At all times, a *p*-value < 0.05 was considered statistically significant. Entire statistical analysis was conducted in STATA version 13.0.

## 3. Results

**Figure 1:**
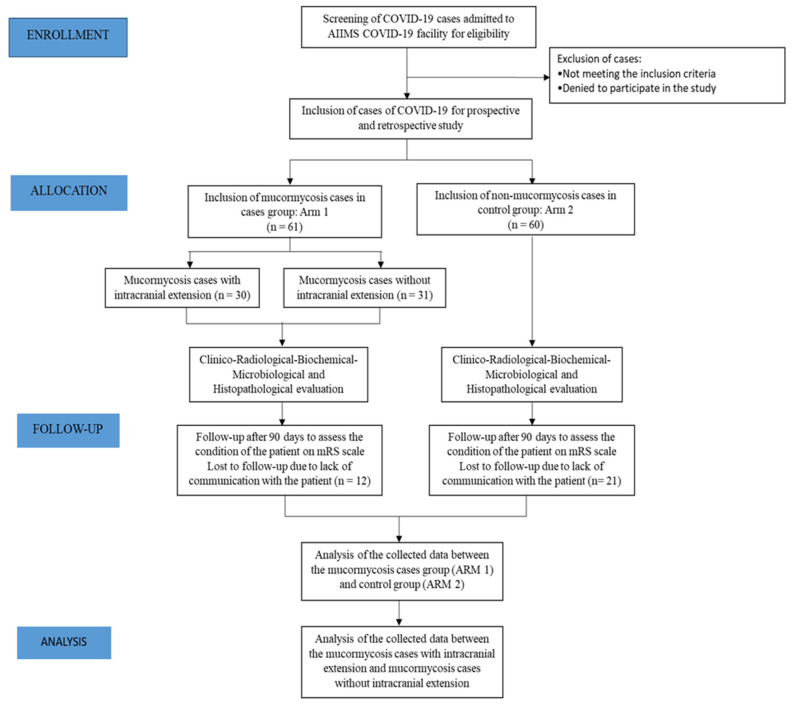
Consort diagram for the study.

A total of 121 patients were included in the study, consisting of 61 COVID-19 patients with mucormycosis (case group) and 60 patients with COVID-19 without mucormycosis (control group) (Figure 1).

**Figure 2:**
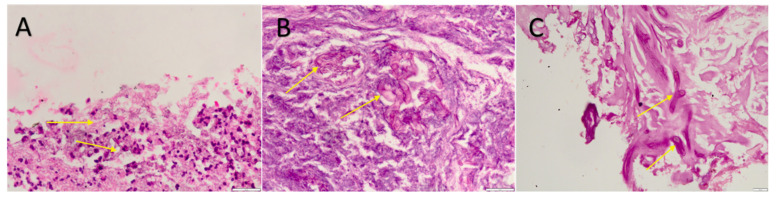
(**A**) Broad aseptate fungal hyphae with numerous neutrophils in background with granulation tissue (**B**) Periodic acidic–Schiff (PAS) stain highlighting the fungal hyphae. (**C**) Numerous broad aseptate fungal hyphae with irregular branching of fungi morphologically suggestive of mucormycosis and background shows necrosis.

COVID-19-associated mucormycosis cases were confirmed by microscopic findings and potassium hydroxide (KOH) staining. Images were obtained from direct microscopic slides suggestive of the gross morphology of the fungal species (Figure 2A–C).

The median (IQR) age of patients in the case group was 49 (43–59) years with 65.6% males and 34.4% females. On the contrary, patients were younger (*p* = 0.0008) in the control group with a median (IQR) age of 38 (29–55.5.) years with 66.6% males. We classified the COVID-19 cases in both the groups, (i.e., cases and controls) into three categories: mild, moderate, and severe as per the guidelines [17]. There were more mild patients (cases 70.9%; controls 60.4%) than the number of moderate patients (cases 21.8%; controls 30.2%), with severe patients comprising the least percentage of total patients enrolled in the study (cases 7.2%; controls 9.3%).

The median duration of symptom onset of mucormycosis from the tested COVID-19 positive was four days (*n* = 53 observations) and inter quartile range (IQR)-2 days to 17 days. The average duration of follow-up of patients was within the range of 90 days to 150 days. The successful number of follow-up cases in the group of mucormycosis cases was 49/61 (80.33%) and in the control group was 39/60 (65%).

The baseline characteristics along with the proportion comparisons are described in Table 1 and symptoms are summarized in Table 2.

Out of all the mucormycosis cases, 100% showed nasal involvement, 41.18% (21/51) patients suffered from rhino-orbital mucormycosis, and 53.57% (30/56) from rhino-orbital-cerebral mucormycosis. Intracranial involvement was observed either in the form of infarct, hemorrhage, meningitis, abscess, or thrombosis (Table 2).

As mentioned before, diabetes mellitus is a major contributing factor in the development of mucormycosis in patients. The frequency of diabetes mellitus in patients in the mucormycosis group (85.7%) was found to be significantly higher than that of patients in the control group (6.9%) (*p* < 0.001). There were more patients with a history of smoking (cases 17.8%; controls 4.6%, *p* = 0.046) in the mucormycosis cases in comparison with the control group (Table 1).

There was a significant association between higher age, diabetes mellitus, oxygen inhalation, a lesser rate of vaccination, higher total leukocyte count (TLC), higher polymorphonuclear leukocytes percentage, higher eosinophils, urea, creatinine, D-dimer, and ferritin levels (Table 1) leading to the occurrence of mucormycosis in cases of COVID-19 affected patients, which have been analyzed in various proposed models using multivariable logistic regression analysis (*p* < 0.05). 

Our study showed more 90-day poor clinical outcomes (mRS three to six) in cases; 32.66% (16/49) higher than in the control group; 5.13% (2/39) (*p* = 0.001) and significantly higher mortality rate in cases; 30.61% (15/49) higher than in the control group; 5.13% (2/39) (*p* = 0.003).

Proportion analysis conducted for poor outcomes after 90 days suggested a significant association of factors such as age, diabetes mellitus, urea, and D-dimer *p* < 0.05) with poor outcomes observed in mucormycosis cases (Table 3).

There were no significant factors that may have led to intracranial involvement in mucormycosis in COVID-19 patients (Table 4).

Prognostic factors such as baseline raised serum creatinine (OR= 4.963; 95% CI 1.456–16.911; *p* = 0.010) and raised D-dimer (OR = 1.00089; 95% CI 1.000097–1.0017; *p* = 0.028) were found to be independent predictors in the prediction model developed using forward stepwise multivariable logistic regression analysis for cases versus controls. (Table 5).

**Figure 3:**
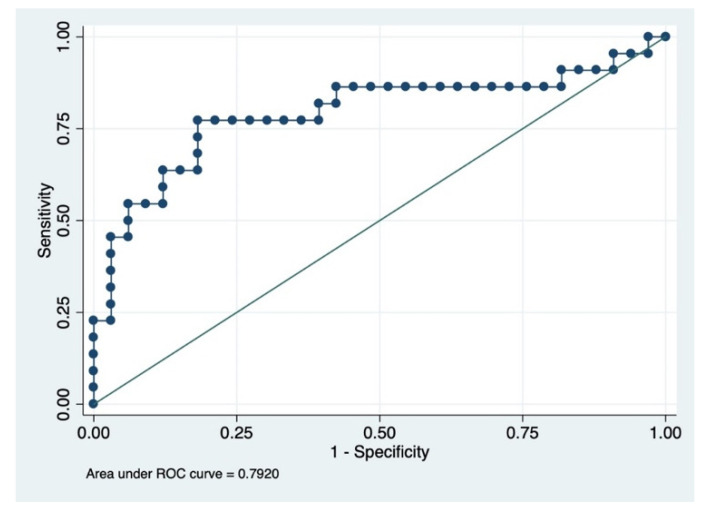
ROC of significant prognostic factors (creatinine and D-dimer) for cases vs. controls. Area under ROC curve = 0.7920 (95% CI = 0.653–0.930).

The prediction model consisting of prognostic factors including baseline creatinine and baseline D-dimer had an area under the ROC curve of 79% (95% CI = 65–93%) (Figure 3).

The model had the following prognostic characteristics: sensitivity = 54.55%, specificity = 90.91%, positive predictive value = 80.00% and negative predictive value = 75.00%. The baseline serum creatinine and D-dimer values were of the para-infective period of COVID-19 (not before developing COVID-19) and not before initiation of the injection of amphotericin B.

Stepwise multivariable analysis prediction model for poor outcome after 90-day follow-up suggested a significant independent association between factors such as steroid usage (OR = 0.070; 95% CI 0.012–0.414; *p* = 0.003), steam inhalation (OR = 0.034; 95% CI 0.003–0.391; *p* = 0.007) and diabetes mellitus (OR = 26.919; 95% CI 1.666–434.892; *p* = 0.020) (Table 5) with poor outcomes in patients.

**Figure 4:**
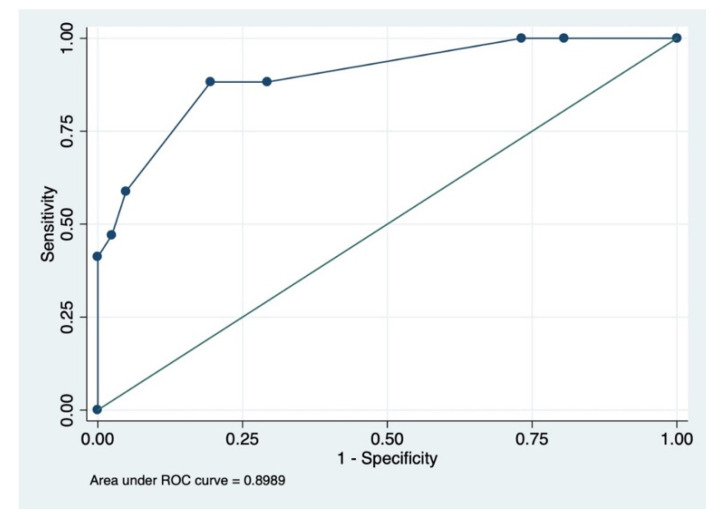
ROC of significant prognostic factors (steroids, steam inhalation and diabetes mellitus) for good outcome vs. poor outcome. Area under ROC curve = 0.8989 (95% CI = 0.811–0.985).

Steroid usage and steam inhalation showed protective effects with respect to the poor outcome in patients, and this discrepancy in the result can be due to the small sample size. The prediction model consisting of prognostic factors including steroids, steam inhalation, and diabetes mellitus had an AUC of 89% (95% CI = 81–98%) (Figure 4).

The model had the following prognostic characteristics: sensitivity = 88.24%, specificity = 80.49%, positive predictive value = 65.22% and negative predictive value = 94.29%. The mortality rate observed in patients with mucormycosis was found to be higher (30.6%) than patients of the control group (5.1%) (OR = 8.162; 95% CI 1.737–38.344; *p* = 0.008).

The prediction model developed after the multivariable analysis for mucormycosis patients with intracranial involvement versus mucormycosis cases without any intracranial involvement, suggested smoking (OR = 0.08; 95% CI 0.009–0.754; *p* = 0.027) as an independent predictor variable (Table 5).

All the patients received an injection of Amphotericin B along with tablets of Posaconazole, and adjunct surgery (where indicated).

## 4. Discussion

To the best of our knowledge, this is the first case–control study reported from a tertiary care hospital in India having 61 cases of mucormycosis with COVID-19 and 60 controls consisting of only COVID-19 patients. Our study showed high levels of baseline raised serum creatinine and raised serum D-dimer in patients with COVID-19 associated mucormycosis as compared to the con-trol group, and diabetes mellitus leading to poor outcomes in cases of mucormycosis with COVID-19 infection.

Various proposed factors which were found to be involved in causing mucormycosis in COVID-19 patients, included diabetes, ketoacidosis, cytokine storm, lymphopenia, high ferritin, endothelialitis, immunosuppression, steam inhalation, poor mask hygiene, contaminated oxygen/humidifiers, excessive antibiotics intake and increased zinc intake [4]. Diabetes mellitus leads to the occurrence of mucormycosis in pre-COVID times as reported in a study with OR 5.5 (*p* = 0.001) [18], while a systematic review reported mucormycosis in the COVID-19 pandemic, with 93% of patients having diabetes mellitus [19,20]. Our findings were in concordance with this published systematic review, as we also observed that 85.71% of patients had diabetes mellitus in cases of mucormycosis with COVID-19.

However, there is no previous study to compare our findings in relation to mucormycosis in cases with COVID-19 infection. We hypothesize a probable association of baseline raised creatinine with the occurrence of mucormycosis in COVID-19 patients. Heightened serum creatinine levels are an indication of renal failure. COVID-19 infections lead to renal dysfunction, which is multifactorial; renal cytotoxicity (SARS-CoV-2 renal tropism), cytokine storm (following macrophage activation due to SARS-CoV-2 and further viral antigen leading to T-helper cell, Th1 and also auto-activation of Th1 cells further causing B cell activation and secretion of various chemokines and cytokines (TNF-α, IL-6, IL-8, IL-21 IL-1β, CCL-2, -3, -5) this inflammatory response leads to multi-organ dysfunction), renal hypoperfusion (fever, diarrhea, vomiting, sepsis, and shock), coagulation abnormalities (raised D-dimer), organ crosstalk due to hypoxia, rhabdomyolysis, cardiac and pulmonary dysfunction [21]. Consequently, renal dysfunction found in COVID-19 patients, an immunocompromised state (reduced levels of CD4^+^ and CD8^+^ cells and increased cytokines circulation) may lead to the occurrence of mucormycosis [22]. Our study also showed reduced absolute lymphocyte count in both mucormycosis and non-mucormycosis COVID-19 patients, although this was a statistically non-significant difference. Chronic kidney disease also leads to increased iron availability in paranasal sinuses and the pulmonary system, which may aggravate the occurrence of mucormycosis [23]. Our study showed the majority of patients who had diabetes mellitus may have associated nephropathy, leading to an increased chance of having mucormycosis in cases with COVID-19. The fungi also grow vigorously in patients with diabetes mellitus who have raised, uncontrolled blood glucose and suffer from diabetic ketoacidosis. The raised glucose concentration and acidosis impede the phagocytic properties of neutrophils, thus weakening the immune response of the patient [13]. Patients with diabetes mellitus have a higher chance of suffering from COVID-19-associated mucormycosis as seen in our study. Raised D-dimer levels were observed in COVID-19 associated mucormycosis patients than in the control group, D-dimer is an indicator of cytokine storm and has an association with thrombotic phenomenon causing stroke, myocardial infarction, venous thrombosis, etc. [24]. There need to be future studies to confirm the association and comprehend the cause–effect relationship of raised creatinine and D-dimer with mucormycosis in patients with COVID-19. In our study, we observed various non-independent factors leading to the occurrence of mucormycosis with COVID-19 viz; higher age, diabetes mellitus, oxygen inhalation, a lesser rate of vaccination, higher total leukocyte count (TLC), higher polymorphonuclear leukocytes percentage, higher eosinophils, urea, creatinine, D-dimer and ferritin levels. On the contrary, we did not find any factors that had an association with the intracranial extension of mucormycosis in patients with COVID-19.

Our study showed a high rate of poor outcomes in cases of mucormycosis with COVID-19; 32.66% as compared to non-mucormycosis cases; 5.13% (*p* = 0.001) and a significantly higher mortality rate in cases of mucormycosis with COVID-19; 30.61% versus 5.13% (*p* = 0.003). The poor outcomes and increased mortality in mucormycosis with COVID-19 infection had an independent association with diabetes mellitus. A study by Dave et al. [25], showed an independent association of diabetes mellitus with unfavorable outcomes and mortality in cases of mucormycosis with COVID-19. This study has reported publications on mucormycosis with COVID-19, according to the study (cases reported > 10) had a mortality of 10% to 40%, which was observed similarly in our study with mortality of 30.61%, while the poor outcomes in our study were observed in 32.66%. There is no case–control study to compare our findings. Our follow-up duration ranged from 90 to 150 days, while reported cases had a mean follow-up duration ranging from 7 to 150 days. Perhaps the duration of follow-up may be extended where more outcomes, such as the recurrence and duration of treatment, could be ascertained [25].

In our study, out of 61 patients, 30 patients had intracranial involvement either in the form of infarct, hemorrhage, meningitis, abscess, or thrombosis. Table 6 describes all the forms of cerebrovascular involvement and reported cases of mucormycosis during the second wave of COVID-19 in India. We noted more mucormycosis in non-vaccinated patients with COVID-19.

There were certain limitations pertaining to this study. Firstly, this is a retrospective study, and therefore recall bias of the patients and selection bias of patients is present. Another limitation was the considerable amount of loss of data due to unsuccessful follow-ups due to the inability to contact the patient. The sample size for the study was also small and therefore the results may not be generalizable. Additionally, the use of steroids and steam inhalation were protective against poor outcomes in patients with mucormycosis with COVID-19. Similarly, smoking was protective against intracranial involvement. This was by chance and may have no clinical significance, and discrepancy in the results can be due to the small sample size and the recall bias of the patients.

## 5. Conclusions

Our study observed elevated levels of baseline raised serum creatinine and D-dimer in COVID-19 associated mucormycosis patients as compared to the control group. Future, larger studies are recommended to establish this cause–effect relationship.

## Figures and Tables

**Table 1:** Baseline characteristics and co-morbidities in cases and control groups.

S. No.	Variable	Cases	Control	*p*-Value
1.	Age in years (Median (IQR))	(58/61) Median (IQR) = 49 (43–59)	(60/60) Median (IQR) = 38 (29–55.5)	0.0008
2.	Sex	Male (38/58) 65.5%	Male (40/60) 66.6%	0.895
		Female (20/58) 34.4%	Female (20/60) 33.3%	
3.	Diabetes	(48/56) 85.71%	(3/43) 6.98%	<0.0001
4.	Hypertension	(17/56) 30.36%	(8/43) 18.60%	0.186
5.	Obesity	(4/25) 16%	(1/43) 2.3%	0.06
6.	Smoking	(10/56) 17.86%	(2/43) 4.65%	0.063
7.	Alcohol	(7/56) 12.5%	(4/43) 9.30%	0.752
8.	Zinc	(8/9) 88.89%	(27/42) 64.29%	0.178
9.	Vitamin C	(12/15) 80%	(38/42) 90.48%	0.299
10.	Steroid	(30/52) 57.70%	(23/43) 53.49%	0.681
11.	Oxygen (In-hospital and/or pre-hospitalization)	(30/56) 53.58%	(14/43) 32.56%	0.039
12.	Steam Inhalation	(40/56) 71.43%	(27/42) 64.29%	0.452
13.	Vaccination	(16/33) 48.48%	(37/42) 80.09%	0.000
14.	Total Leukocyte Count	(55/61) Median (IQR) = 9930 (6200–12010)	(54/60) Median (IQR) = 6720 (5360–10900)	0.009
15.	Polymorphonuclear leukocytes (%)	(55/61) Median (IQR) = 79.5 (69.5–85.8)	(54/60) Median (IQR) = 70.8 (60.3–82)	0.020
16.	Lymphocytes (%)	(55/61) Median (IQR) = 13 (8–20.8)	(54/60) Median (IQR) = 21.25 (9.6–30.2)	0.002
17.	Absolute Lymphocyte Count	(55/61) Median (IQR) = 1153.44 (900.9–1334.64)	(54/60) Median (IQR) = 1174.69 (852.72–2049.52)	0.29
18.	Eosinophils (%)	(55/61) Median (IQR) = 1 (0.4–1.9)	(54/61) Median (IQR) = 0.2 (0–0.8)	<0.0001
19.	Urea	(55/61) Median (IQR) = 38 (23.5–57.8)	(55/60) Median (IQR) = 25 (17–32)	0.0001
20.	Creatinine	(55/61) Median (IQR) = 0.8 (0.64–1.53)	(55/60) Median (IQR) = 0.8 (0.6–0.9)	0.02
21.	Sodium	(55/61) Median (IQR) = 135 (133–138)	(55/60) Median (IQR) = 139 (138–141)	<0.0001
22.	D-dimer	(55/61) Median (IQR) = 396 (264–732)	(50/60) Median (IQR) = 283.30 (251.01–466.59)	0.04
23.	Ferritin	(50/61) Median (IQR) = 1110.5 (529–1650)	(52/60) Median (IQR) = 385.60 (169–741.5)	<0.0001
24.	Procalcitonin	(55/61) Median (IQR) = 0.27 (0.08–2)	(52/60) Median (IQR) = 0.04 (0.02–0.10)	<0.0001
25.	Mortality rate after 90 days	(15/49) 30.61%	(2/39) 5.13%	0.003
26.	Poor outcome (after 90 days)	(16/49) 32.66%	(2/39) 5.13%	0.001

**Table 2:** Percentage of symptoms and various forms of intracranial involvement in patients with COVID-19-associated mucormycosis.

S. No.	Symptoms	Percentage of Patients with the Symptom
1.	Fever	8.93
2.	Headache	44.64
3.	Facial swelling	42.86
4.	Swelling of eyes	51.92
5.	Diminution of vision	38.18
6.	Facial pain	71.43
7.	Eye pain	28.57
8.	Ptosis	9.09
9.	Ulceration	3.57
10.	Nasal discharge	8.93
11.	Facial paralysis	1.79
	Various forms of intracranial involvement	Percentage of patients displaying this form of involvement
1.	Infarct	33.33
2.	Haemorrhage	6.66
3.	Abscess	10.0
4.	Meningitis	6.66
5.	Thrombosis	26.66

**Table 3:** Proportion comparison for poor outcome after 90 days.

S. No.	Variable	Cases	Control	*p*-Value
1.	Age	(16/49) Median (IQR) = 51 (44–66.5)	(2/39) Median (IQR) = 41.5 (20–63)	0.029
2.	Sex	Male (11/16) 68.75%	Male (21/33) 63.64%	0.939
		Female (5/16) 31.35%	Female (12/33) 36.36%	
3.	Diabetes mellitus	(16/16) 100%	(25/33) 75.76%	0.001
4.	Hypertension	(2/16) 12.50%	(14/33) 42.42%	0.38
5.	Obesity	(2/10) 20%	(2/13) 15.38%	0.246
6.	Smoking	(2/16) 12.50%	(6/33) 18.18%	0.99
7.	Alcohol	(2/16) 12.50%	(5/33) 15.15%	0.99
8.	Zinc	(0/0)	(8/9) 88.89%	-
9.	Hydroxychloroquine	(2/5) 40.00%	(4/18) 22.22%	0.93
10.	Steroid usage	(3/15) 20.00%	(23/31) 74.19%	0.002
11.	Oxygen Inhalation	(8/16) 50.00%	(21/33) 63.64%	0.923
12.	Steam Inhalation	(7/16) 43.75%	(31/33) 93.94%	0.001
13.	Haemoglobin	(16/48) Mean = 11.60 ± 1.94	(2/33) Mean = 15.15 ± 2.47	0.374
14.	Total Leukocyte Count	(16/48) Median (IQR) = 11300 (9435–12310)	(2/33) Median (IQR) = 6475 (2050–10900)	0.210
15.	Polymorphonuclear leukocytes	(16/48) Median (IQR) = 79.25 (72.5–86.8)	(2/33) Median (IQR) = 55.65 (19–92.3)	0.99
16.	Lymphocyte	(16/48) Median (IQR) = 10.6 (7.5–20.9)	(2/33) Median (IQR) = 18.8 (4.1–33.5)	0.99
17.	Absolute Lymphocyte Count	(16/48) Median (IQR) = 1234.09 (958.33–1640.77)	(2/33) Median (IQR) = 566.82 (446.9–686.75)	0.05
18.	Monocytes	(16/48) Median (IQR) = 4 (2.55–5.75)	(2/33) Median (IQR) = 6.55 (2.8–10.3)	0.53
19.	Eosinophils	(16/48) Median (IQR) = 0.8 (0.1–1.35)	(2/33) 0.3 (0.1–0.5)	0.32
20.	Platelet	(16/48) Median (IQR) = 236 (155.5–405.5)	(2/33) Median (IQR) = 211.5 (26–397)	0.57
21.	Bilirubin	(16/48) Median (IQR) = 0.52 (0.34–0.69)	(2/34) Median (IQR) = 0.23 (0.2–0.26)	0.10
22.	Alanine transaminase (ALT)	(16/48) Median (IQR) = 21 (17.5–43)	(2/34) Median (IQR) = 12 (9–15)	0.06
23.	Aspartate Aminotransferase (AST)	(16/48) Median (IQR) = 25.5 (23–35.5)	(2/34) Median (IQR) = 16 (13–19)	0.04
24.	Gamma-glutamyl Transferase (GGT)	(16/47) Median (IQR) = 59 (40.5–107)	(2/12) Median (IQR) = 22 (18–26)	0.12
25.	Uric acid	(16/47) Mean = 4.73 ± 1.37	(2/34) Mean = 4.9 ± 1.55	0.109
26.	Urea	(16/48) Median (IQR) = 44.9 (38.5–78.1)	(2/34) Median (IQR) = 29.5 (23–36)	0.09
27.	Creatinine	(16/48) Median (IQR) = 1.2 (0.8–2.3)	(2/34) Median (IQR) = 0.6 (0.5–0.7)	0.07
28.	Sodium	(16/48) Median (IQR) = 133.5 (132.5–138.5)	(2/34) Median (IQR) = 134.5 (131–138)	0.78
29.	Potassium	(16/48) Median (IQR) = 4 (3.5–4.65)	(2/33) Median (IQR) = 4.1 (4–4.2)	0.94
30.	D-dimer 1 (Baseline)	(16/48) Median (IQR) = 394 (278.5–2464)	(2/30) Median (IQR) = 2205 (1171–3239)	0.26
31.	D-dimer 2	(16/48) Median (IQR) = 487.5 (355.5–1043.5)	(2/20) Median (IQR) = 2240.5 (801–3680)	0.18
32.	D-dimer 3	(16/48) Median (IQR) = 623 (356.5–847)	(2/14) Median (IQR) = 2314 (948–3680)	0.08
33.	LDH	(9/35) Median (IQR) = 283 (271–349)	(2/31) Median (IQR) = 257 (182–332)	0.64
34.	Procalcitonin	(16/48) Median (IQR) = 0.16 (0.07–0.43)	(2/31) Median (IQR) = 0.96 (0.75–1.17)	0.12
35.	Ferritin	(15/44) Median (IQR) = 1117 (486.9–1650)	(2/33) Median (IQR) = 1001.5 (602–1401)	0.76

**Table 4:** Proportion comparison for mucormycosis cases vs. mucormycosis cases with intracranial extension.

S. No.	Variable	Mucormycosis Cases with Intracranial Involvement	Mucormycosis Cases	*p*-Value
1.	Age	30/56 Median (IQR) = 46 (44–54)	26/56 Median (IQR) = 52 (43–64)	0.190
2.	Sex	Male (18/30) 60%	Male (18/26) 69.230%	0.473
		Female (12/30) 40%	Female (8/26) 30.769%	
3.	Diabetes	(24/28) 85.7%	(23/26) 88.4%	0.764
4.	Hypertension	(11/28) 39.2%	(6/26) 23.0%	0.204
5.	Obesity	(3/14) 21.4%	(1/11) 9.1%	0.604
6.	Smoking	(2/28) 7.1%	(8/26) 30.7%	0.037
7.	Alcohol	(1/28) 3.5%	(5/26) 19.2%	0.095
8.	Vitamin C	(6/8) 75%	(6/7) 85.7%	0.609
9.	Steroid	(11/25) 44%	(18/25) 72%	0.048
10.	Oxygen	(14/28) 50%	(14/26) 53.8%	0.778
11.	Steam Inhalation	(19/28) 67.8%	(20/26) 76.9%	0.459
12.	Vaccination	(7/16) 43.7%	(9/15) 60%	0.368
13.	Total Leukocyte Count	(28/30) Median (IQR) = 9990 (5830–11820)	(25/26) Median (IQR) = 9040 (6640–11900)	0.89
14.	Polymorphonuclear leukocytes	(28/30) Median (IQR) = 78.25 (69.55–84.5)	(25/26) Median (IQR) = 79 (68.6–83.9)	0.82
15.	Lymphocytes	(28/30) Median (IQR) = 12.25 (8.3–20.2)	(25/26) Median (IQR) = 13 (9–20.8)	0.93
16.	Absolute Lymphocyte Count	(28/30) Median (IQR) = 1126.8 (953.35–1453.57)	(25/26) Median (IQR) = 1155 (844.9–1333.8)	0.71
17.	Eosinophils	(28/30) Median (IQR) = 1 (0.45–2.05)	(25/26) Median (IQR) = 0.9 (0.4–1.5)	0.56
18.	Urea	(28/30) Median (IQR) = 34 (22.45–55.4)	(25/26) Median (IQR) = 38 (30–47)	0.71
19.	Creatinine	(28/30) Median (IQR) = 0.82 (0.62–1.45)	(25/26) Median (IQR) = 0.8 (0.65–1.37)	0.92
20.	Sodium	(28/30) Median (IQR) = 134.5 (132.5–138)	(25/26) Median (IQR) = 136 (133–138)	0.59
21.	D-dimer	(28/30) Median (IQR) = 416.5 (275.5–1563.5)	(25/26) Median (IQR) = 347 (243–721)	0.27
22.	Ferritin	(28/30) Median (IQR) = 973.05 (499.4–1648.5)	(20/26) Median (IQR) = 1116.5 (752.35–1393.85)	0.87
23.	Procalcitonin	(28/30) Median (IQR) = 0.26 (0.08–1.15)	(25/26) Median (IQR) = 0.35 (0.08–2)	0.98
24.	Mortality rate after 90 days	(10/26) 38.4%	(5/21) 23.8%	0.288
25.	Poor outcome (after 90 days)	(11/26) 42.3%	(5/21) 23.8%	0.188

**Table 5:** Univariable and multivariable regression analyses.

S. No.	Variable	Univariable Analysis	Multivariable Analysis
Mucormycosis cases vs. controls			
1.	Creatinine	OR 2.876; 95% CI (1.264–6.542); *p* = 0.012	OR 4.963; 95% CI (1.456–16.911); *p* = 0.010
2.	D-dimer	OR 1.0007; 95% CI (1.0001–1.0012); *p* = 0.018	OR 1.00089l 95% CI (1.000097–1.0017); *p* = 0.028
Poor outcome after 90-day follow-up			
1.	Steroid	OR 0.132; 95% CI (0.034–0.506); *p* = 0.002	OR 0.07; 95% CI (0.012–0.414); *p* = -0.003
2.	Steam Inhalation	OR 0.134; 95% CI (0.043–0.416); *p* = 0.001	OR 0.034; 95% CI (0.003–0.391); *p* = 0.007
3.	Diabetes mellitus	OR 12.741; 95% CI (2.713–59.833); *p* = 0.001	OR 26.919; 95% CI (1.666–434.892); *p* = 0.020
Mucormycosis cases vs. mucormycosis cases with intracranial extension			
1.	Smoking	OR 0.173 (95% CI 0.033–0.912); *p* = 0.037	OR 0.08; 95% CI (0.009–0.754); *p* = 0.027

**Table 6:** Summary of previous studies on mucormycosis in COVID-19 patients.

S No. Author, Year	Age	Gender	Case Description	Investigations	Treatment and Outcome
1. Revannavar SM et al., 2021	Middle-aged	Female	Newly detected DM, left eye ptosis, facial pain—days, fever—3 days, tenderness of all left sinuses, left complete ophthalmoplegia, and reduced visual acuity (6/36).	HbA1c—13.39%, admission glucose 378 mg/dl without ketosis. CT PNS—complete opacification of left ethmoid, maxillary, and frontal sinuses. MRI brain—acute infarct in left parietooccipital lobe with subperiosteal abscess. No ICA thrombus, left ICA showed periarterial inflammation	Conventional amphotericin B for 11 days, aspirin, and FESS. CT showed on follow-up reduction in paranasal sinus opacification.
2. Yukiko Maeda, et al., 2021	73 years		Sudden onset left arm weakness and dysarthria. Two months prior had nausea, vomiting, and diplopia with right-side facial pain. NIHSS 13 Past: uncontrolled DM (HbA1C 9.1%) and chronic sinusitis	CT PNS—Mass in right nasal cavity with bony destruction. MRI—acute infarct right frontal lobe, severe right ICA stenosis with thrombus, also cavernous sinus invasion with mass.	Confirmed mucor by PCR. Treated with amphotericin B, capsofungin acetate, and flucytosine. One year follow patient alive.
3. Vidya Krishna et al., 2021	22 years	Male	BMI-44, hypothyroidism, severe COVID pneumonitis, acute ACA territory infarct	Day 11—CT pulmonary angiogram showed segmental pulmonary embolism, CT Head—right ACA infarct with petechial hemorrhages,	Aspirin, LMWH, HCQs, azithromycin, steroids, hemodialysis, meropenem, teicoplanin, argatroban (on event of PE). Died in day 20 with terminal pericarditis, cardiac tamponade, hypotension. Autopsy—disseminated mucormycosis involving lungs, pericardium, lymph nodes, and brain along with thromboembolism in lung, brain, pharynx, nasal mucosa, and trachea.
4. Nehara HR et al., 2021	59 years	Female	Headache, ptosis, chemosis, ptosis, loss of vision, complete ophthalmoplegia, nasal black discharge, hard palate black crust	PNS—bilateral maxillary, ethmoid, left frontal and sphenoid sinusitis, cavernous sinus thrombosis	Liposomal amphotericin B, antibiotics. Patient died.
	68 years	Female	Headache, facial swelling, ptosis, lid edema, loss of vision, complete ophthalmoplegia nasal black discharge, black crust hard palate	Orbital cellulitis, endophthalmitis, cavernous sinus thrombosis, pansinusitis, multiple lacunar infarcts	Liposomal amphotericin, antibiotics. Patient died
5. Werthman et al., 2020	33 years	Female	Left ptosis proptosis, complete ophthalmoplegia, and altered sensorium with diabetic ketoacidosis	Maxillary and ethmoid sinusitis, MRI brain multiple infarction	Surgical debridement and amphotericin B. Patient died
6. Sen M et al., 2021 N= 2826	285 cases (10.1%) with cavernous sinus thrombosis or invasion 95 cases (3.4 %) with ICA stenosis or occlusion			Hemiparesis, altered consciousness, and focal seizures indicating brain invasion and infarction	Surgery, amphotericin B and posaconazole/isavuconazole
7. Davide cesrati et al., 2020	47 years	Female	Obese women with respiratory problems and COVID 19, worsening respiratory failure, partial left hemispheric syndrome	HRCT scan of her thorax revealed diffuse ground-glass opacities in both lungs, brain CT showed subtle low attenuation within the right insular ribbon and frontal lobe, CT demonstrated large bilateral infarctions of both the cerebellar and cerebral hemisphere	Mechanical thrombectomy could not be performed, patient died after 1 day of witnessing infarctions of both the cerebellar and cerebral hemisphere
8. A patel et al., 2020	observational study with 465 patients.			Rhino-orbital mucormycosis was the most common (315/465, 67.7%) presentation followed by pulmonary (62/465, 13.3%), cutaneous (49/465, 10.5%), and others.	Amphotericin B was the primary therapy in 81.9% (381/465), and posaconazole was used as combination therapy in 53 (11.4%) individuals
9. Rahul Kulkarni et al., 2021	49 patients with cerebrovascular involvement were included. Rhino-orbito-cerebral involvement was the most common form of presentation (98%)			Cerebrovascular involvement was seen in 11.8% patients of COVID-associated mucormycosis patients. Type of stroke—infarct, hemorrhage, abscess	Amphotericin B with oral triazoles
10. Pal et al., 2021	Systematic review with 30 case series/ case reports by pooling data from 99 patients with COVID associated mucormycosis			Diabetes mellitus and hypertension were present in patients as co-morbidities. All of the included patients had a history of COVID-19 infection	Parenteral dexamethasone was most commonly used glucocorticoid. Out of the 96 cases followed up, 33 (34%) were dead, only 63 (66%) patients were alive
11. Garg Deepak et al., 2021	55-year-old Male			End-stage kidney disease, diabetes, COVID-19, and pulmonary mucormycosis. Eight cases of COVID-19-associated mucormycosis included in the review	For treatment received 5 g of liposomal amphotericin B
12. Dilek A. et al., 2021	Case report and systematic review with 30 publications describing 100 patients. 54 years old Male			A 54-year-old male was hospitalized due to severe COVID-19 pneumonia. Common risk factors were corticosteroid use, hypertension, and diabetes.	A 54-year-old male died of sepsis. Death was observed in 33 out of 99 patients. Medical and surgical treatment was given to patients

## Data Availability

The data presented in this study is anonymised. Due to ethical, legal and privacy issues the data is not shared publicly.
